# The predictive role of identifying frailty in assessing the need for palliative care in the elderly: the application of machine learning algorithm

**DOI:** 10.1186/s41043-025-00841-2

**Published:** 2025-04-23

**Authors:** Zahra Nejatifar, Ahad Alizadeh, Mohammad Amerzadeh, Shideh Omidian, Sima Rafiei

**Affiliations:** 1 Student Research Committee, School of Health, Qazvin University of Medical Sciences, Qazvin, Iran; 2 Non-Communicable Diseases Research Center, Research Institute for Prevention of Non-Communicable Diseases, Qazvin University of Medical Sciences, Qazvin, Iran; 3 Medical Microbiology Research Center, Qazvin University of Medical Sciences, Qazvin, Iran; 4 School of Medicine, Qazvin University of Medical Sciences, Qazvin, Iran; 5 Social Determinants of Health Research Center, Research Institute for Prevention of Non-Communicable Diseases, Qazvin University of Medical Sciences, Qazvin, Iran

**Keywords:** Frailty, Palliative care, COPD, Patients

## Abstract

**Background:**

Palliative care is a key component of integrated care to improve care quality and reduce hospitalization costs for patients with chronic obstructive pulmonary disease (COPD). This study aims to use machine learning algorithms to create an effective approach to the early recognition and identification of frailty as a long-term condition in COPD patients.

**Methods:**

The level of frailty in a sample of patients (total n = 140) was assessed using the checklist of frailty assessment, which encompasses five questions: measured decrease in body mass index (BMI), fatigue status, physical activity status, and walking speed. The last question assessed disability through forced expiratory volume in the first second (FEV1) measured using spirometry results. The next checklist was the Palliative Care Needs Assessment Tool, taken from the assessment checklist for palliative care needs in patients with COPD by Thoenesen et al. [28]. We used different machine learning algorithms, with performance assessed using an area under the receiver-operating characteristic curve, sensitivity, and specificity, to develop a validated set of criteria for frailty using machine learning.

**Results:**

Study findings revealed that the palliative care needs assessment tool categorized 74% of all patients into two groups: those requiring palliative care and those not requiring it. Furthermore, the influential variables that contributed to predicting the need for palliative care included measured BMI reduction, fatigue status, physical activity level, slow walking, and FEV1. The super-learning model demonstrated higher accuracy (92%) than other machine-learning algorithms.

**Conclusion:**

The study highlights the need for more collaboration between clinicians and data scientists to use the potential of data collected from COPD patients in clinical settings with the purpose of early identification of frailty as a long-term condition. Predicting palliative care needs accurately is critical in these contexts, as it can lead to better resource allocation, improved healthcare delivery, and enhanced patient outcomes.

## Introduction

The World Health Organization (WHO) defines healthy aging as"the process of developing and maintaining the functional ability that enables well-being in older age"[1]. As global populations age rapidly, the need for tailored healthcare strategies for the elderly has never been more pressing. Iran, one of the fastest-aging countries, is projected to become one of the oldest nations in the world by 2050, surpassing the global average [2, 3]. The WHO refers to this demographic shift in Iran as a"silent tsunami,"urging policymakers to address the emerging challenges associated with an aging population, including the healthcare needs of the elderly [4].

With an increasing elderly population, healthcare systems must adjust to meet their complex needs. This situation presents a significant challenge for developing countries, which may not be fully prepared for the health, medical, social, and economic implications of aging [5]. As a result, the disparity in the quality of life between elderly individuals in developed and developing nations is becoming more apparent, emphasizing the need for proactive strategies to improve the health and well-being of aging populations.

One common and debilitating condition affecting the elderly is chronic obstructive pulmonary disease (COPD) [6]. COPD often manifests in middle age, with its prevalence rising as individuals age [9]. Patients with COPD typically experience persistent symptoms such as mucus production, shortness of breath, chronic coughing, and chest tightness [7, 8]. This chronic condition can have wide-ranging effects on the patient’s physical and psychological well-being, often leading to increased fatigue, sleep disturbances, depression, and anxiety [9, 10]. Furthermore, acute exacerbations of COPD can result in rapid breathing, increased heart rate, excessive sweating, and skin discoloration, negatively impacting the patient's quality of life and daily activities [11]. The long-term nature of this condition, combined with its psychological impact, makes hospitalization both challenging and burdensome for patients and their families [11].

### Theoretical justification: the role of frailty in COPD progression

Frailty, particularly in elderly populations, is recognised as a critical factor in predicting adverse outcomes across a variety of chronic conditions, including COPD. The relationship between frailty and the risk of unfavorable evolution in COPD can be attributed to several interconnected physiological and pathological mechanisms, including global impairment, sarcopenia, and the burden of comorbidities, which collectively exacerbate the progression of COPD [12, 13].

Frailty is often characterised by a state of global impairment involving multiple systems, including musculoskeletal, cardiovascular, and respiratory systems, all of which contribute to worsened COPD outcomes. As frailty encompasses both physical and functional decline, patients may experience diminished exercise tolerance, decreased ability to engage in daily activities, and increased reliance on others for mobility and care. In COPD, this global impairment can lead to a greater risk of hospitalization, respiratory failure, and mortality, as the body’s overall capacity to compensate for pulmonary deficits becomes increasingly limited [12, 13].

Sarcopenia, defined as the progressive loss of muscle mass and strength, is a hallmark of frailty and a significant contributor to COPD progression. Muscle wasting in COPD patients is not only associated with diminished strength but also with decreased respiratory muscle function, impairing the ability to breathe effectively and maintain oxygen levels. Sarcopenia has been shown to correlate with poorer survival rates and higher rates of acute exacerbations in COPD, as it directly impacts the patient's capacity for pulmonary rehabilitation and tolerance to physical activity [14]. Moreover, sarcopenia may also exacerbate symptoms such as fatigue, weakness, and difficulty in mobilizing, further compromising the patient's overall health.

Frail COPD patients often present with a higher burden of comorbidities, including cardiovascular diseases, diabetes, and osteoporosis, which further complicate COPD management and worsen patient prognosis. The presence of these comorbid conditions increases the likelihood of hospital admissions, prolonged recovery times, and increased mortality risks. For example, COPD and heart failure frequently coexist in the elderly, with both diseases contributing to an elevated risk of dyspnea, functional decline, and poor quality of life. The exacerbation of COPD symptoms due to comorbidities may lead to a vicious cycle, where the progression of COPD accelerates frailty, and frailty, in turn, exacerbates the negative outcomes of COPD [15].

Recognizing frailty as a prognostic factor in COPD allows clinicians to identify patients at high risk for poor outcomes, including increased hospital admissions, respiratory failure, and mortality. Studies have demonstrated that frailty indices, which consider components such as sarcopenia, reduced physical performance, and cognitive decline, are strong predictors of survival and functional decline in COPD patients [16]. Therefore, a comprehensive assessment of frailty in COPD patients can serve as an essential tool for predicting clinical trajectories and determining the need for interventions such as palliative care. Early identification of frailty can guide the implementation of tailored management strategies, including physical rehabilitation, nutrition support, and timely palliative care, which may ultimately improve quality of life and reduce unnecessary hospitalizations [17].

While palliative care is well integrated into healthcare systems in countries like England and Canada, its application remains limited in countries like Iran, where resources allocated to healthcare are often insufficient. The lack of infrastructure and awareness surrounding palliative care in these regions underscores the need for more effective and timely strategies to predict when elderly patients, especially those with chronic diseases like COPD, would benefit from palliative care [18]. Predicting palliative care needs accurately is critical in these contexts, as it can lead to better resource allocation, improved healthcare delivery, and enhanced patient outcomes.

### The role of artificial intelligence in predicting palliative care needs

Recent advances in artificial intelligence (AI) have demonstrated significant potential in healthcare, particularly in predicting patient outcomes and needs. AI and machine learning algorithms can process vast amounts of patient data, identifying patterns and predicting the likelihood of clinical events or the need for specific types of care, including palliative care [18]. AI has already been successfully applied in various healthcare settings to predict the progression of chronic diseases, assess patient frailty, and recommend timely interventions [19]. The ability to predict palliative care needs using AI can enhance the decision-making process by enabling healthcare providers to intervene earlier and personalize care plans for patients [20]. For instance, machine learning models have been developed to predict hospital readmissions, disease progression, and the timing of palliative care referrals, based on a combination of clinical data, patient history, and real-time health information [21, 22]. These algorithms have shown promise in improving patient outcomes by enabling timely interventions and reducing unnecessary hospitalizations. Furthermore, AI-driven systems have the potential to optimize healthcare resources, making the provision of palliative care more efficient and cost-effective [23].

In the context of COPD, AI can be particularly valuable. By analysing longitudinal data from electronic health records (EHRs), sensor data, and patient-reported outcomes, AI can identify patients at risk of exacerbations and deterioration in their condition, flagging those who may benefit most from palliative care [24]. This predictive capability can help clinicians prioritize care, ensuring that patients receive the right care at the right time.

This study proposes a novel model based on the Superior Learner model, a machine learning framework, to predict the palliative care needs of elderly patients with COPD. The integration of AI into this process not only enhances the accuracy of identifying patients who would benefit from palliative care but also optimizes healthcare delivery in resource-limited settings like Iran. By predicting these needs with greater precision, we aim to reduce unnecessary hospitalizations, enhance the quality of life for patients, and maximize the efficiency of healthcare resource utilization.

## Materials and methods

### Study participants

The demographic and clinical data of 140 COPD patients referred to the respiratory clinic in a tertiary medical center in Qazvin, Iran from August to November 2023 was collected. In this study, we used Green’s formula to estimate the minimum sample size required for the study. Green’s formula (25) offers an approximate calculation for determining the minimum sample size necessary for conducting a multiple regression analysis. The formula is expressed by the following equation:

N ≥ 50 + 8⋅m.

Where

N = Minimum required sample size;

m = Number of predictors (independent variables) in the model.

In this study, drawing on similar research, we included 10 samples for each checklist indicator being examined. Considering that the performance and accuracy of predictive models and machine learning algorithms increase with the number of recorded samples, this study follows the pattern of similar studies and considers ten samples for each study indicator [25].

### Patient recruitment procedure

Eligible participants were identified from the clinic’s database. Upon arrival at the clinic, potential participants were approached by the research team and informed about the purpose of the study. They were provided with detailed information regarding the study procedures, the objectives of the research, and what their participation would involve. If they expressed interest in participating, they were given time to ask questions and decide whether they wished to proceed.

Written informed consent was obtained from all participants before any data collection began. The consent form outlined the nature of the study, the use of personal health information for research purposes, and the voluntary nature of participation. Participants were informed that they could withdraw from the study at any time without affecting their medical care. The informed consent procedure was carried out by a member of the research team who ensured that all participants fully understood the information provided.

Patient privacy and confidentiality were strictly maintained throughout the study. All data collected was anonymised and stored in secure systems. The study complied with all relevant national and international ethical standards to ensure that the participants'rights were protected.

The study protocol was reviewed and approved by the Ethical Review Committee of the medical center (Approval number: IR.QUMS.REC.1402.105). All research procedures were carried out in accordance with the principles of the Declaration of Helsinki and relevant ethical standards for medical research.

### Measures

The features used in machine learning were the need for palliative care as an outcome variable and a set of input variables, including a decrease in BMI, fatigue status, physical activity status, and walking speed representing the frailty level. Furthermore, a variable representing disability among patients was assessed through forced expiratory volume in the first second (FEV1) measured using spirometry results. Each patient profile also included some demographic and clinical variables, including age, gender, marital status, smoking status, drug and alcohol consumption, and blood pressure.

### Definition of frailty and cut-off points

Frailty was assessed using a frailty assessment tool consisting of five questions measuring key frailty indicators: decrease in BMI, fatigue status, physical activity, walking speed, and forced expiratory volume in the first second (FEV1/FVC) measured by spirometry. Patients were classified as"frail"if they exhibited three or more of these frailty indicators, following the general criteria used in previous studies. This classification aligns with the conceptualization of frailty as a state of global impairment that encompasses multiple domains, including physical and functional decline, weight loss, and reduced lung function [26]. The cut-off points for each scale were based on established thresholds in the literature and clinical guidelines:

*BMI Decrease* A decrease of 5% or more in body mass index (BMI) within the last six months.

*Fatigue* Patients were considered fatigued if they reported significant fatigue (a score above a predefined threshold on the fatigue scale).

*Physical Activity* Patients reporting a decrease in physical activity were classified as frail if they scored below the threshold defined by the Short Physical Performance Battery (SPPB) scale.

*Walking Speed* A walking speed of less than 0.8 m/s was considered indicative of frailty.

*FEV1/FVC* A forced expiratory volume in one second to forced vital capacity ratio below 0.7 was used to define disability related to COPD.

### Data collection tools

We used a frailty assessment tool with five questions to gather patient data around variables, including measured decrease in BMI, fatigue status, physical activity status, and walking speed. The last question assesses disability through FEV1/FVC using spirometry results. Patients were classified as frail if they exhibited three or more of these parameters, while those with fewer than three were considered healthy and normal in terms of pulmonary function [27].

While the Fried Frailty Phenotype has been extensively validated in Western populations, its adaptation to Iranian settings has been limited. However, a study by Ahmadi et al. [26] evaluated the reliability and validity of a Persian version of the Fried Frailty Phenotype in Iranian elderly populations and found it to be a suitable tool for assessing frailty [26]. Minor linguistic modifications were made to ensure clarity for Persian-speaking patients. Additionally, since hand-grip strength is less frequently assessed in Iranian clinical settings, we substituted FEV1/FVC as an objective measure of physical impairment, a method previously validated in COPD research [27].

Another checklist was the Palliative Care Needs Assessment Tool, derived from the Thoenesen et al. study for evaluating palliative care needs in COPD patients [28]. This six-item checklist encompasses various criteria, including evaluating disability status through the Karnofsky score, which measures patient dysfunction, monitoring for a 10% weight loss over the preceding six months, identifying the presence of congestive heart failure, assessing for orthopnea, probing the patient's outlook on life to gauge if they perceive themselves as nearing the end of life and scrutinizing objective symptoms of significant breathlessness. To determine the need for palliative care, a minimum of three indicators from this assessment checklist must be present to initiate the palliative care phase [28].

The original checklist by Thoenesen et al. was developed in a Western healthcare context. Since no formally validated Persian version was available, the checklist was translated into Persian and reviewed by a panel of pulmonologists and palliative care specialists in Iran to ensure content validity and cultural relevance. The Karnofsky Performance Scale (KPS) has been previously validated in Iranian cancer patients [28], and its use in COPD patients was deemed appropriate.

### Machine learning algorithms

Various machine learning methods were used for this purpose, including the Bayesian Generalized Linear Model,^1^ Random Forest, Generalized Additive Models,^2^ Support Vector Machine,^3^ Bayesian Additive Regression Trees,^4^ Kernel k-nearest Neighbors, and average. In the first step, data was collected through interviews and patient assessments, and incomplete data was excluded. Then, the remaining data was categorized into two groups: training and testing. Using the training data, each of the mentioned models was trained.

*Bayesian Generalized Linear Models* It's a robust statistical approach that integrates two key statistical methodologies: the general linear model and Bayesian inference. It serves as a potent instrument in data analysis and forecasting dependent variables, facilitating high accuracy and interpretability by utilizing plausible and understandable parameter estimates and predictions [1].

*Random Forests* This is an influential machine-learning technique employed for classification and regression tasks. Through the amalgamation of numerous decision trees, this model demonstrates resilience against overfitting to training data and can effectively handle incomplete and noisy datasets. Additionally, by aggregating the outcomes of individual trees, the model's accuracy and predictive capabilities can be enhanced [2].

*Generalized Additive Model* This is a machine learning approach employed to capture the correlation between a dependent variable and one or multiple independent variables. This model amalgamates nonlinear and elementary linear functions and can adjust to input data by employing versatile functions, such as smooth and basic functions [3].

*Support Vector Machines* This algorithm is among the machine-learning techniques utilized for classification and regression tasks. It can capture both linear and nonlinear associations within data. The regression support vector machine endeavors to identify the optimal level for interpreting the data by leveraging support vectors [4].

*Bayesian Additive Regression Trees* This machine learning model is applied to regression tasks and text generation challenges. Employing the Bayesian learning method, trees within the Elliptic Tree Machine model are constructed, utilizing the concept of incrementally adding new trees to the model set. This iterative addition of trees enhances the model's capacity to learn intricate and broadly applicable relationships between input and output variables [5].

*Generalized Linear Models* This statistical approach is applied to analyze continuous data and distributions that deviate from normality. The model serves as an extension of the linear model, enabling its adaptation to non-normal distributions. Within this framework, the relationship between the dependent variable and independent variables is expressed through a function known as the link function. Leveraging the link function, the model accommodates various distributions, including normal, binomial, Poisson, and gamma distributions [6].

*Kernel k-Nearest Neighbor* This machine learning model is employed in classification and regression tasks. It builds upon the k-NN algorithm and incorporates a kernel function to compute the similarity among samples. Utilizing a kernel function in the Generalized Linear Model and k-NN with kernel function offers the advantage of assessing the similarity between samples in a non-linear manner. This capability enables the model to capture more intricate relationships between attributes and outputs [7].

*Super Learner Model* This model combines several ensemble learning models and provides a robust approach to predicting highly nonlinear, complicated problems. Van et al. proposed an ensemble learning algorithm, which improves the reliability and accuracy of the model by combining different models such as GBM, AdaBoost, CatBoost, LightGBM, and GLM as a meta-learner (52 super). The ensemble learning methods have become popular as they gain more reliable results than single prediction models (Lee). Moreover, it is essential to provide the values of different ensemble learning methods, which assists researchers and AI developers optimize the models and achieve high reliability and accuracy [8].

### Handling of missing data

When data were missing for less than 10% of any variable, it was handled through imputation using the mean for continuous variables (e.g., age, BMI) or the mode for categorical variables (e.g., gender, marital status, smoking status). For variables with more than 10% missing data, the respective records were excluded from the analysis to maintain the validity of the results. In total, 5% of the data was excluded due to missing values in key variables, particularly from participants who were unable to complete the required assessments (e.g., spirometry tests or frailty assessment items).

### Data analysis

To develop and evaluate the predictive model, we utilized the Super Learner library in R version 4.3.2. The dataset was split into training (80%) and testing (20%) sets to ensure that the model was validated on unseen data.

### Internal validation approach

To enhance the generalizability of the model and mitigate the risk of overfitting, we implemented tenfold cross-validation on the training data. This method involved:

- Randomly dividing the training dataset into 10 equal subsets (folds).
- Using 9 folds for training and onefold for validation in each iteration.
- Repeating the process 10 times, ensuring each fold served as validation once.
- Averaging the results across all iterations to obtain a robust performance estimate.

The Area Under the Curve (AUC) of the Receiver Operating Characteristic (ROC) curve was the primary metric used to compare model performance. A higher AUC indicated a superior ability to distinguish between patients needing palliative care and those who did not.

### Super learner execution and convergence criteria

The Super Learner model combined multiple base learners, including Bayesian Generalized Linear Models, Random Forest, Generalized Additive Models, Support Vector Machines, Bayesian Additive Regression Trees, Kernel k-nearest Neighbours, and Generalized Linear Models.

The Super Learner algorithm was run for 100 iterations to optimize the model weights assigned to each base learner. The optimization process terminated when the cross-validated risk (mean squared error for regression or log loss for classification) stopped improving beyond a predefined threshold (0.001). A Generalized Linear Model (GLM) with a logit link function was used as the meta-learner to combine the predictions from the base learners.

## Results

Findings of the descriptive statistics section revealed that men constituted the largest statistical population of this study, 76 percent. In terms of marital status, 83% of patients were married, and 17% were single. Furthermore, 85% of the participants were unemployed or retired. In terms of smoking status, 82% of people were not currently smokers, and only 18% were smokers, but 57% of people had a history of smoking in the past. Thirteen percent of COPD patients used at least one type of drug. In terms of alcohol consumption, 19% of patients stated that they had a history of use. Finally, regarding clinical condition, 56% of study participants had a history of high blood pressure, 23% had a history of diabetes, and 31% had a history of high blood lipids (Table 1).

**Table 1:** Demographic, Lifestyle, and Clinical Information of Study Participants

		Frequency	Percentage
Gender	Woman	33	24
	Man	107	76
Marital status	Single	24	17
	Married	116	83
Employment status	Unemployed	119	85
	Employed	21	15
Current smoking status	Smoker	25	18
	Non-smoker	115	82
Past smoking status	Smoker	80	57
	Non-smoker	60	43
Drug use status	Yes	18	13
	No	122	87
Alcoholic beverages use status	Yes	27	19
	No	113	81
History of high blood pressure	Yes	78	56
	No	62	44
History of diabetes	Yes	32	23
	No	108	77
History of high blood lipids	Yes	43	31
	No	97	69

Additionally, the demographic, lifestyle, and clinical characteristics of the patients, categorized by their frailty status, are presented in Table 2.

**Table 2:** Patient’s Characteristics based on their Frailty Condition

Characteristics		Total (mean ± SD)	Patients with Frailty (mean + SD)	Patients without Frailty (mean + SD)	p-value
Age		71.34±6.23	73.54±7.39	69..83±4.77	0.001
BMI		23.46±3.67	21.59±3.48	24.75±3.22	0.001
Marital status	Female	(17.14%) 24	(29.82%) 17	(8.43%) 7	0.002
	Male	(82.86%) 116	(70.18%) 40	(91.57%) 76	
Income	Good	(%7.86) 11	(%1.75) 1	(12.05%) 10	0.006
	Moderate	(46.43%) 65	(38.60%) 22	(51.81%) 43	
	Weak	(45.71%) 64	(59.65%) 34	(36.14%) 30	
Smoking	Yes	(%82.14) 115	(%89.47) 51	(77.11%) 64	0.074
	No	(17.86%) 25	(10.53%) 6	(22.89%) 19	
Drug use	Yes	(87.14%) 122	(80.70%) 46	(91.57%) 76	0.072
	No	(12.86%) 18	(19.30%) 11	(8.43%) 7	
Alcohol consumption	Yes	(80.71%) 113	(87.72%) 50	(75.90%) 63	0.118
	No	(19.29%) 27	(12.28%) 7	(24.10%) 20	
History of hypertension	Yes	(%44.29) 62	(51.81%) 43	(%33.33) 19	0.029
	No	(55.71%) 78	(48.19%) 40	(66.67%) 38	
History of diabetes	Yes	(77.14%) 108	(70.18%) 40	(81.93%) 68	0.143
	No	(22.86%) 32	(29.82%) 17	(18.07%) 15	
History of blood lipids	Yes	(69.29%) 97	(59.65%) 34	(75.90%) 63	0.053
	No	(30.71%) 43	(40.35%) 23	(24.10%) 20	

According to Table 2, patients with frailty were significantly older than those without frailty (*P* = 0.001). Furthermore, frailty was associated with lower BMI, with frail individuals showing significantly lower values (*P* < 0.001). Likewise, marital status and income level were significantly related to frailty (*P* = 0.002), with lower-income individuals experiencing higher frailty rates. Hypertension history was another factor that significantly associated with frailty (*P* = 0.029), with 51.81% of frail patients having a history of high blood pressure.

In the next step, we examined the predictive role of frailty components on the need for palliative care among COPD patients. Findings revealed that patients experiencing frailty had a 13.09 times higher likelihood of requiring palliative care.

Furthermore, according to Table 3, BMI reduction, when controlling for the effects of other factors, could significantly affect the need for palliative care (*P*-value < 0.001**)**. Fatigue and physical activity status had also a marginal effect on the outcome variable with relatively 0.063 and 0.066 *P*-values. Slow walking and FEV1/FVC were not significantly associated with frailty (*P*-value = 0.136, and 0.162). However, all five factors were statistically significant in the univariate analysis.

**Table 3:** Frailty Components as Predictors of the Need for Palliative Care

Frailty Factors	Multivariable Analysis		Univariate Analysis	
	OR (95% CI)	P-value	OR (95% CI)	*P*-value
Reduction in BMI	(3.37,28.91)9.81	< 0.001	14.4 (6.09–37.88)	< 0.001
Feeling of Fatigue	(0.96,8.04)2.76	0.063	8.57(3.77,20.40)	< 0.001
Physical Activity Level	3.54(0.92,13.46)	0.066	12.8(5.47–33.58)	< 0.001
Slow walking	0.37(0.10,1.33)	0.136	0.06(0.03,0.15)	< 0.001
FEV1/FVC	2.62(0.67,10.62)	0.162	5.17(1.84,18.53)	0.004
Frailty	–	–	13.09 (4.19–41.07)	< 0.001

**Fig. 1:**
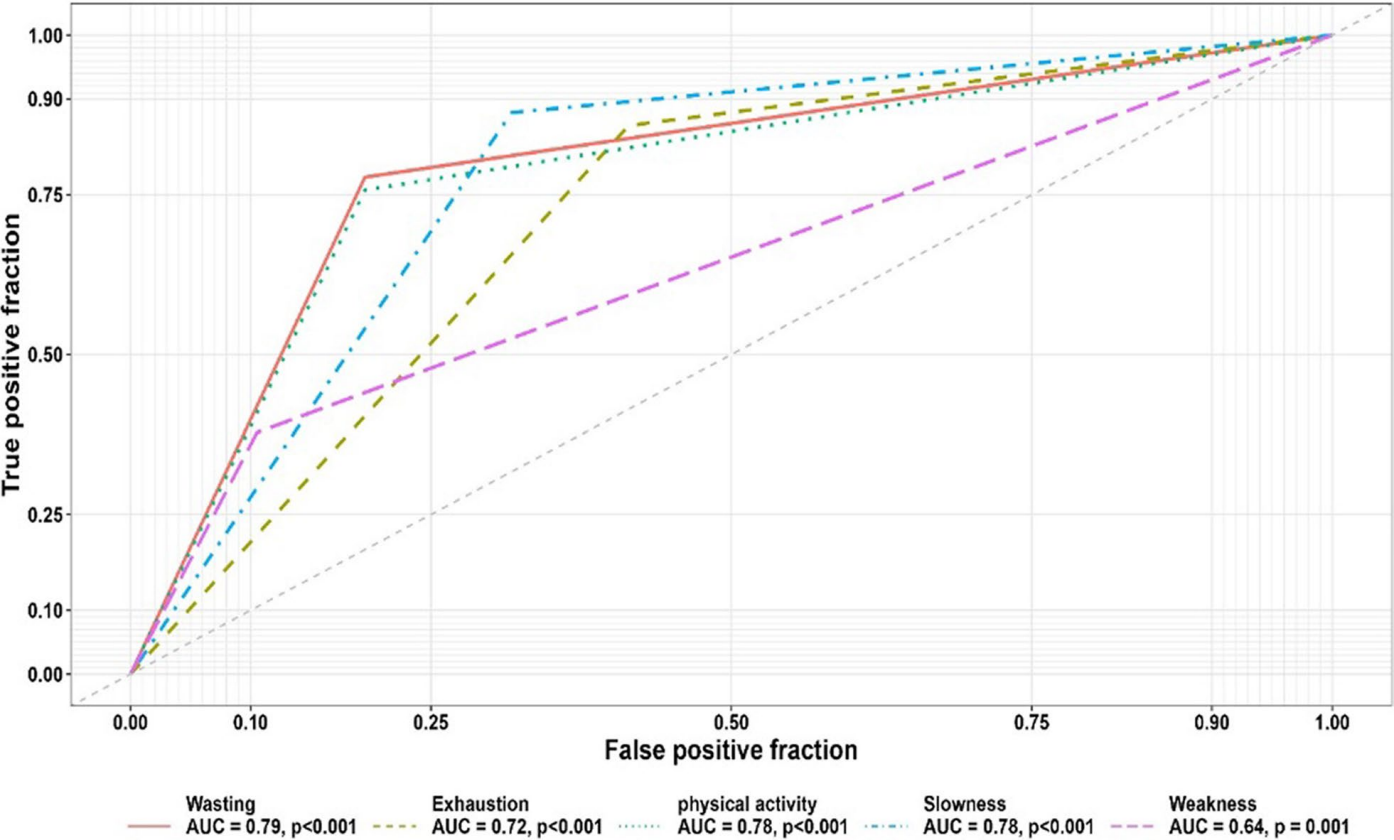
The area under the curve (AUC) percentages for different frailty components

Figure 1 depicts the accuracy rate of predictions for the need for palliative care based on each of frailty components. According to the Area Under the Curve (AUC) percentages, a reduction in BMI demonstrated an acceptable discriminatory ability in distinguishing between the two patient groups: those requiring palliative care and those not requiring it. This suggests that reduction in BMI can effectively help differentiate patients who may benefit from palliative care services with an accuracy rate of 79%. Physical activity and slow walking were two other predictors representing a model with acceptable discriminatory ability followed by FEV1/FVC with the lowest discriminatory ability.

In machine learning, particularly when evaluating predictive models, sensitivity and specificity are key performance metrics that help assess the effectiveness of a model in identifying the different classes. Table 4 depicts the specificity and sensitivity of frailty factors in predicting the need for palliative care. A high sensitivity value means the model is good at identifying the patients who need palliative care (true positives), reducing the risk of missing those who should receive it. Meanwhile specificity measures the proportion of actual negatives (patients who do not need palliative care) correctly identified by the model.

**Table 4:** Specificity and Sensitivity of Frailty Factors in Predicting the Need for Palliative Care

Frailty Factors	Cut-off point	Specificity	Sensitivity
Reduction in BMI	0.91 (0.92, 0.92)	80.49(68.29,92.68)	77.78(69.69,85.85)
Feeling Fatigue	0.54(0.54, 0.54)	58.54(43.90,73.17)	85.86(78.78,91.91)
Physical Activity Level	0.94(0.95, 0.95)	80.49(68.29,92.68)	75.76(66.66,83.83)
Slow walking	0.53(0.53, 0.53)	68.29(53.65,80.48)	87.88(80.80,93.93)
FEV1/FVC	1.19(1.18,1.18)	89.47(78.94,97.36)	37.80(28.4,47.56)

According to the table, BMI reduction with a sensitivity of 77.78% indicates that nearly 78% of patients who truly need palliative care are accurately identified as needing it. The specificity of 80.49% for this measure shows that 80.49% of patients who do not need palliative care are correctly identified as not requiring it. Among the various factors, slow walking demonstrated the highest sensitivity, followed by fatigue. In contrast, the highest specificity was associated with the FEV1/FVC ratio, followed by physical activity and BMI reduction. The high specificity and low sensitivity of the FEV1/FVC ratio suggest that the model excels at correctly identifying patients who do not need palliative care but may miss many patients who do require it, resulting in a higher number of false negatives.

### Clinical implication of FEV1/FVC performance

The high specificity but low sensitivity of the FEV1/FVC ratio suggests that it is useful for confirming when a patient does not need palliative care but less effective at detecting those who do. This is expected, as spirometry measures like FEV1/FVC primarily assess airflow limitation rather than frailty-related functional decline. In clinical practice, this implies that while FEV1/FVC can help rule out patients who are not at risk, it should not be used as the sole determinant for identifying those in need of palliative care. Instead, frailty measures and BMI reduction should be prioritized for screening.

Finally, we combined different AI algorithms and used a super learner model to achieve the best possible prediction. According to Table 5, the Bayesian generalized linear model exhibited a prediction power of 0.283, and the Random Forest model had a prediction power of 0.168; while the performance of generalized linear model, support vector machine, and generalized linear regression tree was zero, indicating no significant impact on predicting the need for palliative care.

**Table 5:** Performance of Different AI Models

Model	Performance
Bayesian Generalized Linear Model	0.283
Random Forest	0.168
GAM	0
SVM	0
BART	0
GLM	0
Kernel k-NN	0.474
Average	0.073

These findings highlight that kernel k-NN was the most effective individual model for predicting palliative care needs.

**Fig. 2:**
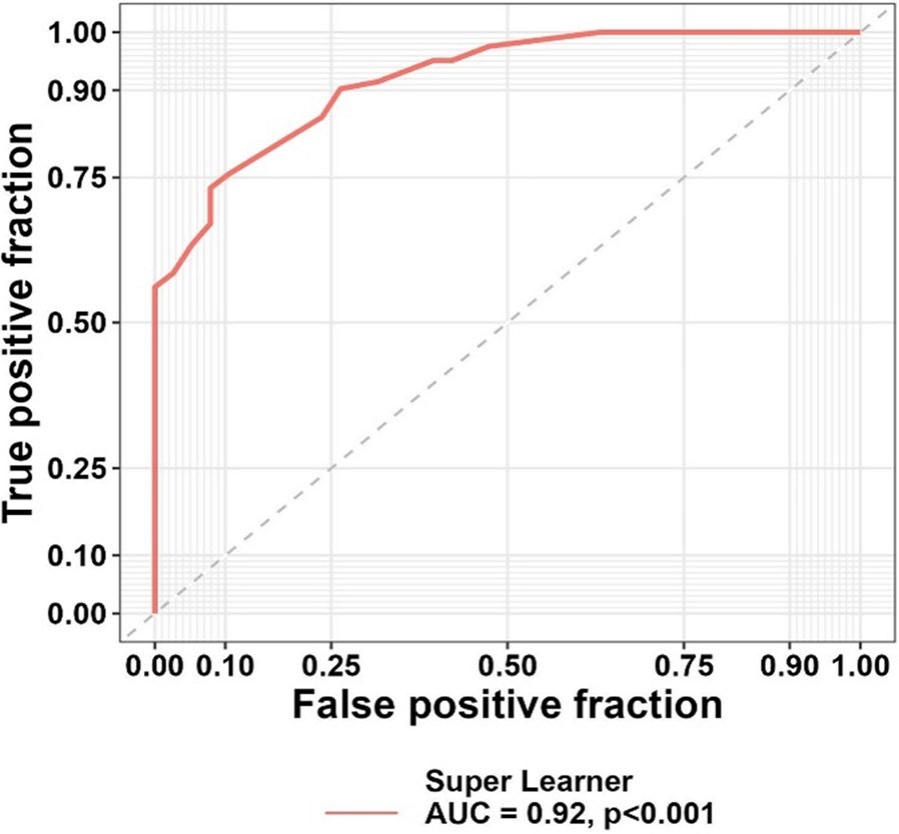
The predictive power of the super learner model

Using the super learner model, we integrated the models mentioned in the above table and plotted a ROC curve to provide the best combination for predicting the need for palliative care. ROC curve analysis in Fig. 2 demonstrates that the new model correctly classified 92% of all patients into two groups: those requiring palliative care and those not requiring it (*P*-value < 0.001).

Figure 2 illustrates the ROC curve of the Super Learner model, which achieved an AUC of 92% (*P* < 0.001), indicating excellent predictive performance.

As shown in Table 6, using the super learner model, 91.46% of individuals who required palliative care were correctly identified, and 89.47% of patients who did not need such services were accurately distinguished. The cutoff point is 0.59, indicating that individuals with a score higher than 0.59 require palliative care.

**Table 6:** Specificity, Sensitivity and F1 score of the Super Learner Model

	Cut-off point	Specificity (%)	Sensitivity (%)	F1 score
Farilty questionnaire	0.59	89.47	91.46	0.983

These results confirm that the Super Learner model significantly improves predictive accuracy, surpassing individual AI algorithms.

## Discussion

This study aimed to develop a predictive model using the super learner approach to identify palliative care needs in elderly patients with COPD. Our findings highlight that frailty, as assessed by a dedicated questionnaire, was able to successfully classify 74% of COPD patients into two groups: those requiring palliative care and those not requiring such services. Key variables influencing palliative care needs included reductions in BMI, fatigue status, and physical activity level. These results align with a growing body of literature emphasizing the significance of frailty and other clinical markers in predicting palliative care requirements in COPD patients.

### Comparison with existing literature

Our results are consistent with previous studies that underscore the role of frailty in predicting adverse outcomes for COPD patients. For instance, Marengoni et al. [29] conducted a systematic review and found that frailty assessment tools, such as the Cardiovascular Health Study Frailty Phenotype and the Frailty Index, effectively predicted mortality and hospitalizations in COPD patients [29]. Similarly, Maddocks et al. [30] reported that the Fried Frailty Phenotype was significantly associated with increased mortality and readmission risks following acute exacerbations in COPD patients [30]. Lahousse et al. [31] also demonstrated that frailty, as measured by the Frailty Index, was independently linked to higher all-cause and respiratory-related mortality risks in COPD patients [31]. Moreover, Bernabeu-Mora et al. [32] validated the SHARE Frailty Instrument as an effective tool in identifying frailty and its association with mortality and hospitalization risks in this population [32].

In the realm of machine learning, our study's findings are comparable to those of Blanes-Selva et al. [33], who successfully used a random forest model to predict the need for palliative care in COPD patients using clinical and demographic factors [33]. Our enhanced predictive model using the super learner approach demonstrates the added value of ensemble methods in healthcare predictions. By combining multiple models, our study supports the argument that ensemble techniques can significantly improve predictive accuracy, a concept that has also been highlighted in the broader machine learning literature.

### Predictive power of the super learner model

Our study adds to the growing body of research demonstrating the value of ensemble methods, particularly the super learner approach, in clinical prediction tasks. In our model, the integration of multiple algorithms significantly enhanced predictive accuracy compared to standalone models. This improvement reflects the strengths of ensemble learning, which leverages the diversity of individual algorithms to improve the robustness and generalizability of predictions. This finding is consistent with other studies in healthcare, which have shown that ensemble methods outperform single algorithms in various clinical prediction tasks [34, 35].

The super learner model's ability to classify 92% of patients correctly into two groups—those requiring palliative care and those not needing such services, highlights its potential for practical application in clinical settings. The fact that all five checklist factors (BMI reduction, fatigue, physical activity, and others) were significant predictors of palliative care needs further supports the effectiveness of our predictive model. In line with existing literature, the use of multi-faceted data, including clinical, demographic, and functional information, improves the accuracy of predictions regarding palliative care needs [32–35].

### Impact of key variables on palliative care needs

Among the key variables identified, BMI reduction, fatigue status, and physical activity level emerged as significant predictors of palliative care needs in COPD patients. These findings align with existing research linking low BMI with increased mortality and poor prognosis in COPD patients. For example, Luo et al. [36] reported that low BMI is associated with higher mortality and hospitalization risks in COPD patients, underscoring the potential need for palliative interventions [36]. Weight loss in COPD often signifies disease severity, muscle wasting, and systemic inflammation, all of which contribute to increased palliative care needs.

Similarly, a systematic review by Bellolio et al. [37] emphasized that fatigue is a common and debilitating symptom in COPD, and its presence is a strong indicator of the need for palliative care [37].

Physical activity has also been consistently identified as a critical factor influencing the prognosis of COPD patients. Moy et al. [38] demonstrated that low levels of physical activity are linked to increased mortality and healthcare utilization, which supports the need for palliative care in such individuals [38]. Despite the well-documented associations between physical activity and health outcomes, our study did not find a significant association between slow gait speed and frailty. This finding differs from research by Kon et al. [39], which identified slow gait speed as a predictor of mortality and exacerbations in COPD patients [39]. This discrepancy may be attributed to differences in study design, sample size, or measurement tools used, highlighting the complexity of predicting palliative care needs based on single markers like gait speed.

### Limitations of FEV1 as a sole predictor

While many studies, including Celli et al. [12], have identified low FEV1 as a key predictor of mortality in COPD patients, our study did not find a significant association between FEV1 and frailty. This finding is consistent with the argument that FEV1 alone may not be a reliable marker for predicting palliative care needs. Several studies have suggested that other factors, such as functional status, fatigue, and BMI, may provide a more comprehensive understanding of COPD prognosis [12]. This reinforces the idea that the multifactorial nature of COPD requires a more holistic approach to identifying patients in need of palliative care.

### Implications for clinical practice

Our findings suggest that by combining frailty assessments with machine learning models, healthcare providers can significantly improve their ability to predict palliative care needs in COPD patients. The super learner model offers a promising tool for clinical decision-making. By incorporating diverse predictors such as BMI, fatigue, and physical activity, the model provides a nuanced and comprehensive approach to identifying patients who may benefit from palliative interventions.

As shown in studies by Almagro et al. [40] and Avati et al. [41], integrating disease-specific variables into predictive models can enhance their ability to forecast patient outcomes. These models, including our super learner approach, can guide clinicians in making timely and accurate decisions about palliative care referrals, potentially improving patient outcomes and reducing unnecessary hospitalizations. Furthermore, research by Downer et al. [42] has demonstrated that predictive models for palliative care can outperform traditional clinical decision-making, reinforcing their utility in real-world clinical settings.

### Study limitations

One challenge encountered was the lack of comprehensive information in patients'medical records. To mitigate this, we conducted face-to-face interviews and carefully reviewed all available medical data to ensure thorough data collection. Another limitation was the difficulty in accessing a validated checklist for assessing palliative care needs. To address this, we performed extensive literature searches and contacted authors of relevant studies to obtain the necessary tools for our analysis. Third, the relatively small cohort may limit the generalizability of our findings. Therefore, larger, multicenter studies are needed to validate the predictive performance of the Super Learner model. Fourth, the absence of external validation is another limitation of the study that might restrict the broader applicability of our model. Future research should focus on validating our findings in diverse populations to assess their robustness and clinical utility. Lastly, while our model incorporated key clinical markers, additional factors such as socioeconomic status, medication adherence, and psychological status may further refine predictive accuracy.

Despite these limitations, this study’s innovative use of advanced machine learning techniques, its comprehensive approach to identifying palliative care needs, and its high predictive accuracy make it a standout contribution to the field of COPD and palliative care research. These strengths highlight the potential for future clinical applications, setting the stage for improved patient outcomes and better-targeted palliative care interventions.

## Conclusion

In conclusion, this study supports the use of advanced machine learning techniques, specifically the super learner approach, to predict palliative care needs in COPD patients. By integrating multiple predictive models, we achieved a higher level of accuracy in identifying patients who would benefit from palliative care interventions. Our results are consistent with existing literature and provide additional evidence of the utility of machine learning models in clinical practice. The identification of key factors, such as BMI reduction, fatigue, and physical activity, reinforces the importance of a comprehensive, multifaceted approach to patient care. These findings not only align with existing research but also pave the way for future innovations in predictive modeling that could transform the management of COPD and other chronic conditions. Ultimately, this study emphasizes the need for precision in identifying palliative care needs, offering a promising avenue for improving patient outcomes and optimizing resource allocation in healthcare settings. The combination of frailty assessments with machine learning tools holds significant promise for improving palliative care decision-making in COPD and other chronic conditions. Future research should focus on validating these models in larger, diverse populations and further exploring their applicability in routine clinical practice.

## Data Availability

The datasets used and/or analyzed during the current study available from the corresponding author ‎on reasonable ‎request. The entire dataset is in Farsi language. The Data can be available in English ‎language for the readers and make ‎available from the corresponding author on reasonable request.‎

## Footnotes

1 GLM.

2 GAM.

3 SVM.

4 BART.
